# Attentional Selection of Social Features Persists Despite Restricted Bottom-Up Information and Affects Temporal Viewing Dynamics

**DOI:** 10.1038/s41598-018-30736-8

**Published:** 2018-08-22

**Authors:** Aleya Flechsenhar, Lara Rösler, Matthias Gamer

**Affiliations:** Department of Psychology, Julius Maximilian University of Würzburg, Würzburg, Germany

## Abstract

Previous studies have shown an attentional bias towards social features during free-viewing of naturalistic scenes. This social attention seems to be reflexive and able to defy top-down demands in form of explicit search tasks. However, the question remains whether social features continue to be prioritized when peripheral information is limited, thereby reducing the influence of bottom-up image information on gaze orienting. Therefore, we established a gaze-contingent viewing paradigm, in which the visual field was constrained and updated in response to the viewer’s eye movements. Participants viewed social and non-social images that were randomly allocated to a free and a gaze-contingent viewing condition while their eye movements were tracked. Our results revealed a strong attentional bias towards social features in both conditions. However, gaze-contingent viewing altered temporal and spatial dynamics of viewing behavior. Additionally, recurrent fixations were more frequent and closer together in time for social compared to non-social stimuli in both viewing conditions. Taken together, this study implies a predominant selection of social features when bottom-up influences are diminished and a general influence of social content on visual exploratory behavior, thus highlighting mechanisms of social attention.

## Introduction

Amongst the variety of information in the environment, our visual system selects relevant aspects to attend in order to reduce the complexity of incoming input. This allocation of attention is commonly accomplished via eye movements and the method of eye tracking has therefore been used extensively as a straight-forward measure to investigate attentional exploration of naturalistic scenes. To predict gaze patterns and explain their underlying mechanisms, several algorithms have been implemented on the grounds of physical saliency (for review, see Borji & Itti^1^). The majority of these approaches rests on the assumption that high local contrast in visual features (e.g., color, intensity, spatial frequency) should be conspicuous to the viewer and correspondingly attract attention. Indeed, such algorithms performed well in predicting human fixations for a multitude of stimuli under free-viewing conditions^2,3^.

While such saliency approaches particularly emphasize stimulus-driven, bottom-up attentional control, free-viewing entails the engagement of both bottom-up, as well as goal-directed (top-down) attentional processes^4,5^. In our daily lives, however, we do not only freely perceive our surroundings, but often have a certain question in mind – these task-related requirements are known to engage mainly top-down control^6^. Within top-down driven models, a different approach by Najemnik & Geisler^7^ has taken locations of maximum information gain into consideration, characterizing the ideal observer model and emphasizing the role of the resolution of the visual system, which is maximal at the point of the fovea and limited in the periphery. Similarly, Foulsham & Underwood^2^ and Tatler & Vincent^8^ have emphasized the importance of systematic tendencies of eye movements in scenes that may predict gaze behavior as well as saliency models, ensuing that eye movements and attention are associated, as they are driven by the same internal mechanism (see “pre-motor theory of attention”^9^). Importantly, while stimulus-driven and goal-driven attention are closely intertwined in free-viewing conditions, gaze-contingent viewing offers the possibility to effectively restrict pre-attentively available feature information^10^. When only the currently fixated location is revealed to observers, low-level features of the image periphery cannot attract the observers’ eyes in a bottom-up fashion as proposed by saliency models of attention. Indeed, search time, saccade length and fixation durations were found to be affected during gaze-contingent viewing, indicating that differential attentional mechanisms are employed during image exploration^10^. Previous studies have used gaze-contingent viewing windows to investigate how information is acquired during reading^11^ and which field of view optimizes picture memorization^12^. Despite different tasks at hand, both studies rested on the assumption that gaze-contingent windows are moved in such a manner that task execution is optimized. Saccades, however, also tend to process information within the current viewing window as vertical windows trigger a higher number of vertical saccades while horizontal shapes yield more horizontal saccades^13^. It could therefore be argued that gaze-contingent viewing reduces bottom-up processing of peripheral information but cannot entirely eliminate bottom-up processing of stimuli presented within the viewing window.

Nonetheless, Kennedy and Adolphs^14^ demonstrated that gaze-contingent viewing can be used to effectively alter the balance between bottom-up processing and top-down control in order to reveal mechanisms of social perception. They first showed that patient S.M., who suffers from a bilateral amygdala lesion, failed to fixate the eyes of faces when allowed to freely explore the stimuli. However, when viewing the same stimuli through a gaze-contingent window, she exhibited regular eye fixations. This result suggests that gaze-contingent viewing meaningfully eliminates competing bottom-up features of social information which drive gaze behavior. To what extent does gaze-contingent viewing alter gaze patterns when viewing complex naturalistic social scenes? Typically, social features are prioritized over competing physically salient objects when viewing complex naturalistic scenes^15–18^. Specifically, Rösler, End & Gamer^18^ have shown that attention to social features takes place reflexively as revealed by the direction of first saccades after a very brief stimulus presentation time of only 200 ms. While bottom-up processes thus seem to drive social attention, top-down processes, e.g. attempting to spot a friend in a crowded bar, are likely to additionally impact gaze behavior. Flechsenhar & Gamer^17^ showed that the implementation of tasks that specifically intended to drive attention away from social aspects of the scene still resulted in preferential allocation of attention onto depicted human beings. Collectively, these studies suggest that bottom-up mechanisms are essential in driving social attention. However, the precise role of top-down attentional control is less clear since the vast majority of studies in this domain used free-viewing conditions that do not permit a dissociation between bottom-up and top-down processes.

To investigate influences of bottom-up and top-down mechanisms in more detail, the current study contrasted a free-viewing and a gaze-contingent condition. In order to evaluate gaze pattern differences between these conditions more elaborately, we employed recurrent quantification analysis (RQA) which has been previously used to exhibit altered scanpaths depending on stimulus type in a gaze-contingent compared to a free-viewing condition^19^. While Anderson and colleagues^19^ showed increased fixation recurrences in gaze-contingent viewing of naturalistic scenes, it remains unknown whether this increase persists using social stimuli. The aim of the current study was hence two-fold. Firstly, contrasting gaze-contingent with free-viewing conditions, we aimed to investigate top-down influences on social attention when bottom-up visual information is restricted. We expected these top-down mechanisms to manifest in a strong prioritization of social features within the gaze-contingent condition, which would suggest an additional importance of top-down mechanisms in regulating social attention. Secondly, we explored the temporal dynamics of social attention more generally using RQA. Here, we expected to find more recurrent and deterministic fixations for social features supporting the attentional bias towards social information in naturalistic scenes.

## Results

### Saliency-based prediction of fixations

**Figure 1:**
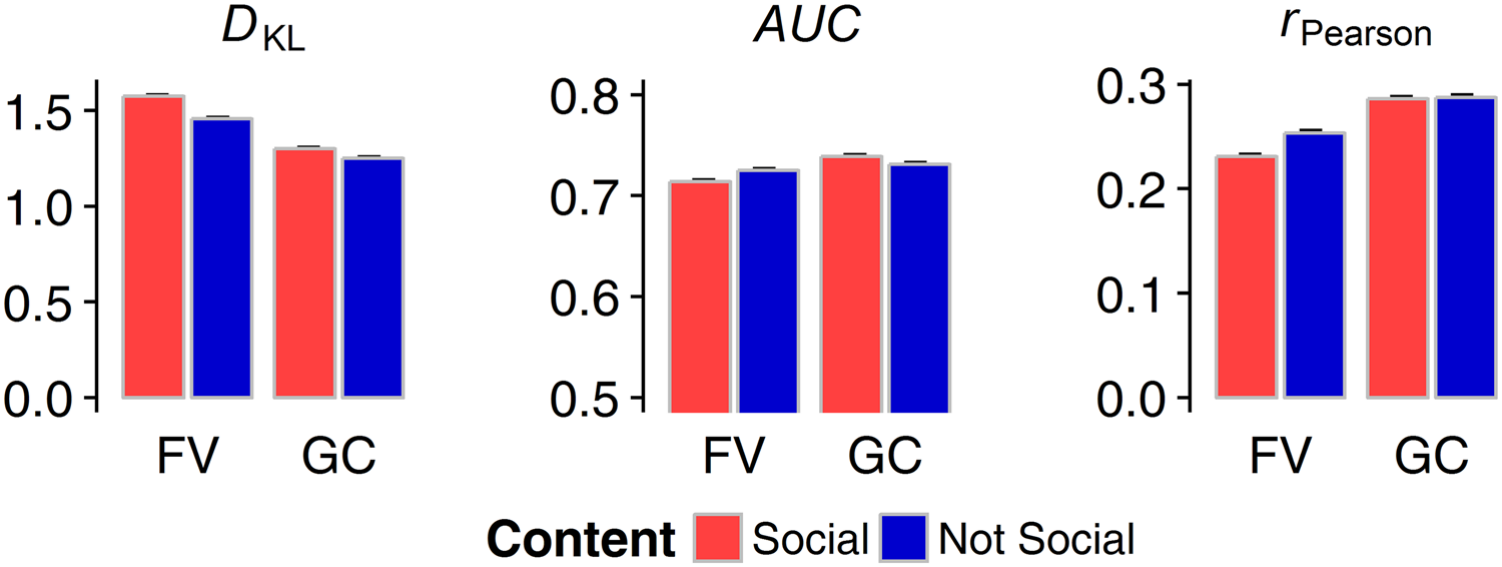
Divergence (Kullback-Leibler divergence, *D*_*KL*_) and correspondence (area under the receiver-operating curve, *AUC*; Pearson product-moment correlation coefficient, *r*) between saliency and fixation density maps for social and non-social scenes in free-viewing (FV) and gaze-contingent (GC) conditions. Error bars represent standard errors of the mean.

As a difference measure between two probability distributions, we analyzed the Kullback-Leibler Divergence (*D*_*KL*_) to examine how well physical saliency predicted the observed eye movements during free and gaze-contingent viewing. Herein, the distributions of saliency and fixations diverged significantly more for social stimuli as compared to non-social ones, as described by a significant main effect of stimulus content (*F*_(1,74)_ = 180.05, *p* < 0.001, η^2^ = 0.031). Further, a significant main effect of viewing condition (*F*_(1,74)_ = 58.29, *p* < 0.001, η^2^ = 0.205) generally describes lower predictability of fixations by saliency in free-viewing than in gaze-contingency. A significant interaction effect of both factors (*F*_(1,74)_ = 30.49, *p* < 0.001, η^2^ = 0.005) refers to smaller differences between stimulus categories within the gaze-contingent condition compared to free-viewing. Coherently, when regarding results for the area under the receiver-operating curve (*AUC*), we found an inverse relationship, namely a significant main effect of viewing condition (*F*_(1,74)_ = 10.05, *p* = 0.002, η^2^ = 0.041) with worse saliency-based prediction of fixations for gaze-contingent displays than for free-viewing. A significant interaction between viewing condition and stimulus category (*F*_(1,74)_ = 41.82, *p* < 0.001, η^2^ = 0.017) describes the observation that fixation predictions were worse for social stimuli in the free-viewing condition, yet better in the gaze-contingent condition. The main effect of stimulus category, however, was not statistically significant (*F*_(1,74)_ = 1.71, *p* = 0.19, η^2^ < 0.001). Results of the Pearson product-moment correlation coefficient (*r*) showed worse saliency-based prediction of fixations for social as compared to non-social stimuli (main effect of stimulus category: *F*_(1,74)_ = 38.52, *p* < 0.001, η^2^ = 0.015), and a significant difference between viewing conditions (main effect of viewing condition: *F*_(1, 74)_ = 45.86, *p* < 0.001, η^2^ = 0.183). Similar to the analysis of *D*_*KL*_, the difference in predictability between stimulus categories was higher in the free-viewing than in the gaze-contingent presentation (interaction effect: *F*_(1,74)_ = 21.81, *p* < 0.001, η^2^ = 0.013) (Fig. 1).

### ROI-Analysis

**Figure 2:**
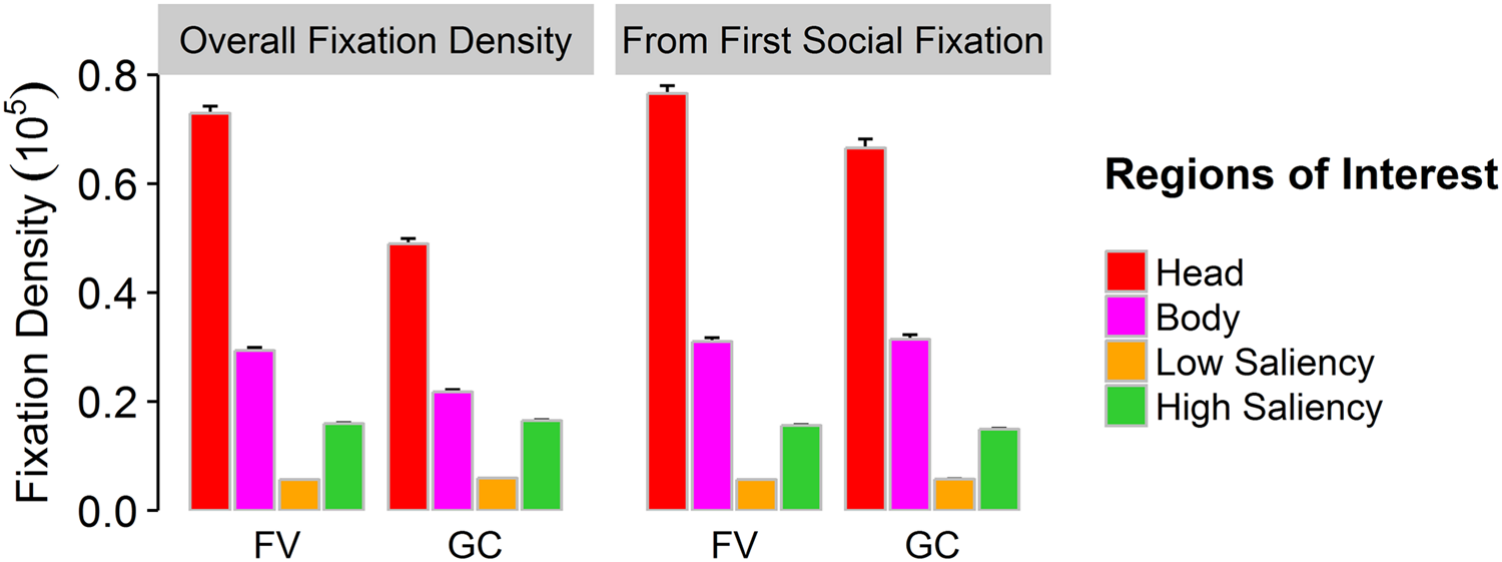
Relative area-normed fixation density on regions of interest (ROIs) for free-viewing (FV) and gaze-contingent (GC) viewing. The left panel depicts the overall fixation densities for the presentation duration of 10 s. The right panel shows fixation densities measured from the time point in which the participants first fixated a social feature until the end of the presentation time of 10 s. Error bars represent standard errors of the mean.

Considering fixation density on pre-defined ROIs, we found a significant main effect of ROI (*F*_(3,222)_ = 1251.166, ε = 0.43, *p* < 0.001, η^2^ = 0.881) with social ROIs gaining most attention, especially heads, compared to all other regions in both conditions. A significant main effect of viewing condition (*F*_(1,74)_ = 166.89, *p* < 0.001, η^2^ = 0.209) describes higher fixation densities in general for free-viewing. We also observed a significant interaction of viewing condition and ROI (*F*_(3,222)_ = 131.33, ε = 0.46, *p* < 0.001, η^2^ = 0.307) depicting overall lower fixation densities for gaze-contingent displays than for free-viewing, which is especially the case for head and body ROIs. The interaction effect may therefore be driven mainly by the fact that exploration of social ROIs is reduced in gaze-contingent displays compared to the free-viewing condition (Fig. 2, left panel). To test whether this may arise from the fact that social stimuli could not be immediately attended due to the masking, we reanalyzed the data starting from the time point at which the social aspect was first fixated.

Indeed, when comparing the time points of first fixations on social ROIs for both viewing conditions, we found that participants needed significantly less time until encountering a social ROI in free-viewing (*M* = 894.88 ms, *SD* = 268.55 ms) than in gaze-contingency (*M* = 2153.76 ms, *SD* = 461.66 ms; *t*_(74)_ = 22.27, *p* < 0.001, *d* = 3.45). When further analyzing fixation data from the time point of this first social detection until the end of the presentation time, we again obtained significant main effects of ROI (*F*_(3,222)_ = 1119.81, ε = 0.41, *p* < 0.001, η^2^ = 0.851) depicting a fixation bias for social ROIs, and a main effect of viewing condition (*F*_(1,74)_ = 7.42, *p* = 0.008, η^2^ = 0.015) implying higher fixation densities for free-viewing as opposed to gaze-contingent viewing. A significant interaction of ROI by viewing condition (*F*_(3,222)_ = 11.23, ε = 0.46, *p* < 0.001, η^2^ = 0.041) emphasizes that fixation densities were different across ROIs and viewing conditions, showing slightly reduced viewing behavior for social ROIs in the gaze-contingent displays. However, compared to the pattern found previously, the difference between free-viewing and gaze-contingency regarding the fixation of social ROIs seems to be slightly smaller (Fig. 2).

### Recurrent Quantification Analyses

**Figure 3:**
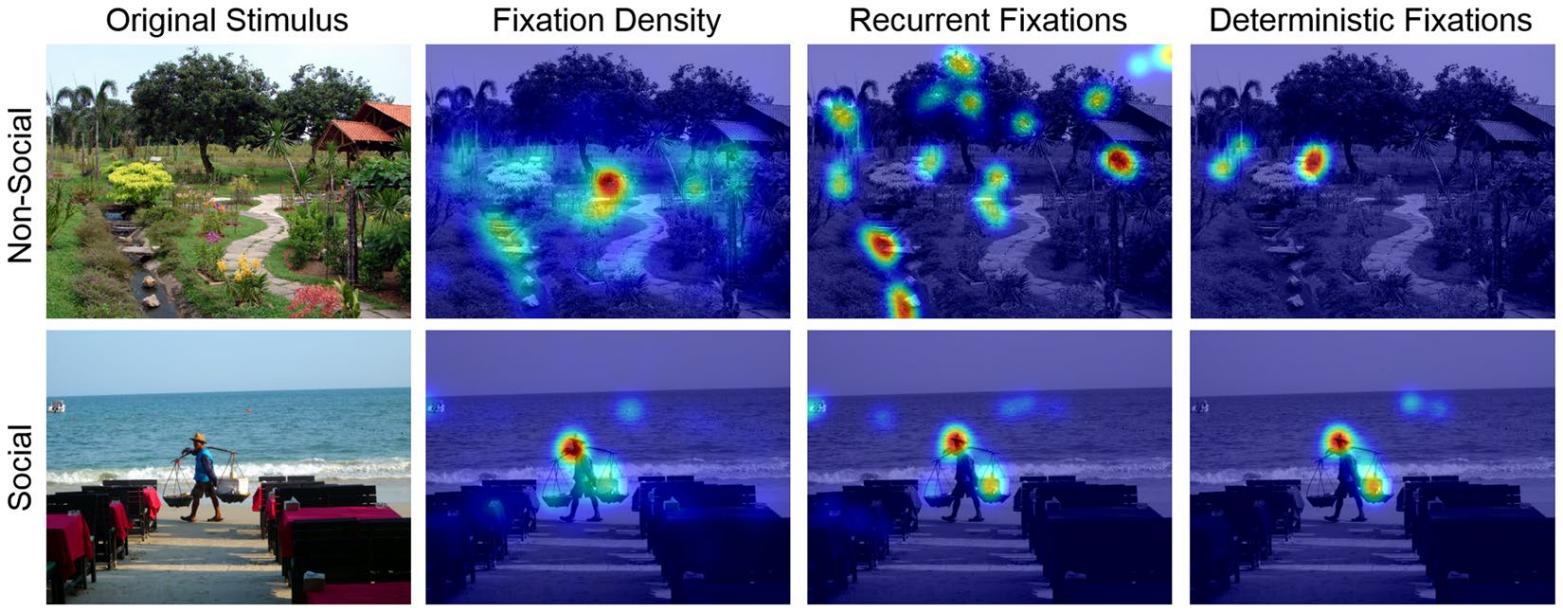
Example of a non-social (top) and a social (bottom) stimulus with respective heat maps for fixation densities, recurrent fixations and deterministic fixations of all participants across both viewing conditions. Warm colors represent areas with higher values of the respective measure, whereas cool colors indicate low values. Image taken with permission from the Nencki Affective Picture System^37^.

Recurrent quantification analyses (RQA) were suggested to complement analyses of fixation density since they provide additional information on the temporal dynamics of fixations. Figure 3 demonstrates that recurrent and deterministic fixations reveal discrepancies to fixation densities that might systematically differ between social and non-social stimulus content.

**Figure 4:**
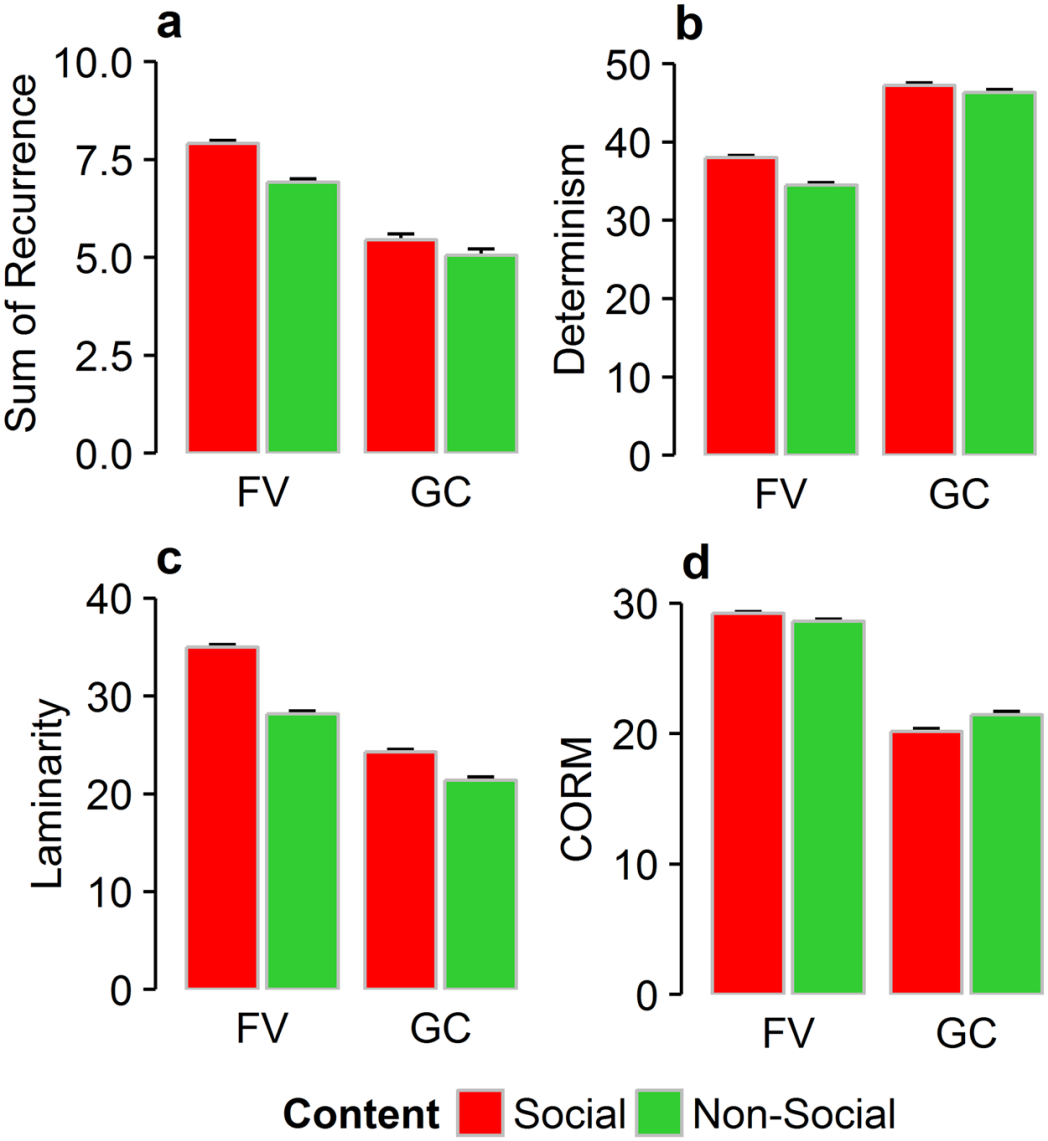
Averages of four recurrence quantification analysis measures: (**a**) Recurrence, (**b**) Determinism, (**c**) Laminarity and (**d**) Center of Recurrent Mass across free-viewing (FV) and gaze-contingent (GC) conditions for social and non-social stimulus content. Error bars represent standard errors of the mean.

In order to systematically quantify the influence of viewing conditions and stimulus category, we analyzed four different RQA measures (see Fig. 4): (1) for the sum of recurrent fixations, we obtained a significant main effect of condition (*F*_(1,74)_ = 31.96, *p* < 0.001, η^2^ = 0.124), indicating higher mean recurrence for the free-viewing condition than for the gaze-contingent display. A significant main effect of content (*F*_(1,74)_ = 104.40, *p* < 0.001, η^2^ = 0.013) describes higher mean recurrence for social than for non-social stimuli. However, we did not find a statistically significant interaction of condition and content (*F*_(1,74)_ = 2.88, *p = *0.09, η^2^ < 0.001), which signifies that there was no significant difference in the sum of recurring fixations between social and non-social stimuli across viewing conditions. (2) Deterministic fixations displayed a reversed pattern with higher means for gaze-contingent than for free-viewing (*F*_(1,74)_ = 97.43, *p* < 0.001, η^2^ = 0.275). Repeated subsequent fixations were also more frequent for social than for non-social stimuli (*F*_(1,74)_ = 46.95, *p* < 0.001, η^2^ = 0.025) but this difference between stimulus content was more pronounced for free-viewing as compared to gaze-contingent viewing as indicated by a significant interaction effect (*F*_(1,74)_ = 10.47, *p = *0.002, η^2^ = 0.005). (3) Laminarity is another fixation repetition measure describing the tendency to attend certain locations multiple times (here more than twice). Our results showed a significant main effect of condition (*F*_(1,74)_ = 121.25, *p* < 0.001, η^2^ = 0.307) with higher laminarity for free-viewing than gaze-contingent viewing and a significant main effect of content (*F*_(1,74)_ = 304.56, *p* < 0.001, η^2^ = 0.111) depicting higher mean values for social stimuli. A significant interaction between condition and content (*F*_(1,74)_ = 32.79, *p* < 0.001, η^2^ = 0.014) suggests that in images with social content locations were revisited more often than in images with non-social content in free-viewing, but less so in gaze-contingent viewing. (4) The measure for center of recurrent mass (CORM) enabled us to examine the temporal distribution of recurrent fixations. A significant main effect of condition (*F*_(1,74)_ = 270.02, *p* < 0.001, η^2^ = 0.474) describes that recurrent fixations were closer in time for gaze-contingent displays than for free-viewing. A significant main effect of stimulus content further shows that recurrent fixations were closer in time for social than for non-social stimuli (*F*_(1,74)_ = 4.16, *p* = 0.04, η^2^ = 0.003). A significant interaction (*F*_(1,74)_ = 14.00, *p* < 0.001, η^2^ = 0.011) between viewing condition and content suggests that recurrent fixations occurred closer in time for social stimuli than for non-social ones in free-viewing, but farther in time for the gaze-contingent condition (Fig. 4).

## Discussion

This study used a gaze-contingent display to investigate social attention when peripheral visual information is limited. The current results from a relatively large group of participants revealed a robust attentional exploration of social features even when reducing the influence of bottom-up mechanisms. Additional analyses of the temporal dynamics of fixation patterns demonstrated increased recurrences and deterministic fixations for social as compared to non-social images which suggests that social information might be special regarding its influence on the generation of priority maps for attentional selection.

In detail, our results showed that social features, especially faces, were preferentially fixated over physically salient areas independent of the viewing condition. Since gaze-contingent paradigms subdue bottom-up driven mechanisms and rely more heavily on voluntary control over gaze direction and allocation, the prevailed, yet somewhat diminished fixation density on social features in our study suggests that social attention involves voluntary attentional selection. When further comparing modalities from the time point at which the first fixation on social features was registered, this disparity across conditions decreased, yet remained significant. This proposes the possibility that the difference in fixation density is partly impacted by the time spent searching for a social element in the gaze contingent condition. Importantly, this attention bias for social features cannot be ascribed to the fact that these aspects were physically highly salient. Consequently, the power of saliency-based predictions was considerably reduced when social features were present in complex naturalistic visual input. This is in line with findings of End & Gamer^16^, who also observed that the influence of physical saliency on gaze behavior is weakened by social stimuli in free-viewing. This further implies that physical saliency is insufficient in predicting gaze behavior when the visual field contains social information^20–23^.

As an investigation of the temporal dynamics of fixation sequences complements the analysis of mere fixation densities, we also examined recurrence quantification measures in both viewing conditions. The characterization of viewing behavior concerning recurrent fixations aimed to find not only differences between viewing conditions, but we were also interested whether viewing dynamics are affected by stimulus content, most importantly with respect to social features. On a general level, our results replicated those of Anderson and colleagues^19^ who observed increased recurrences when natural scenes were viewed freely compared to when they were viewed gaze-contingently. We were further able to replicate the observation that deterministic fixations (i.e., one fixation repeatedly following another) occur more frequently in gaze-contingent viewing, likely due to the sequential targeting of features within the gaze-contingent window. Similarly, we also found laminarity and center of recurrence mass to be increased in free-viewing, suggesting that single fixations were repeated more often and that repetitions generally occurred further apart in the trial sequence in free-viewing than in the gaze-contingent condition. Importantly, although Anderson and colleagues^19^ did use different sets of stimuli (exteriors, interiors and landscapes), our stimuli allowed us to compare re-fixations in social versus non-social scenes to investigate the role of social content in attentional control. This revealed that recurrences were higher for social than for non-social images. Herein, all fixation repetition measures (sum of recurrence, determinism, laminarity) indicated greater recurrences for social than non-social image areas. Furthermore, recurrences were closer together in time for social than non-social image areas as measured by the center of recurrence mass. Conclusively, the results of the recurrence quantification analysis support preferential viewing behavior towards social information shown by fixation densities, by revealing that this prioritization manifests through multiple re-fixations throughout the viewing time.

The combination of viewing modalities allowed an additional examination of predominant top-down control (gaze-contingent viewing) and both, bottom-up and top-down influences (free-viewing) on social attention. While bottom-up processing is not completely eliminated in the gaze-contingent viewing condition, fewer low-level salient information is available near the current fixation and no such details are visible in the periphery. Thus, most executed saccades will draw on top-down processes for the determination of saccade endpoints. Our current results therefore suggest that social attention is not merely reflexive but also relies on top-down attentional processes. So how does social attention then fit into the traditional dichotomy of bottom-up and top-down mechanisms? The recurrence quantification analysis used here further implicates that viewing behavior towards social stimuli is different than for non-social stimuli with regard to fixation sequence as well as temporal structure. Foulsham & Kingstone^24^ already showed that gaze patterns can change with image content in a scene, but our data presents explicit differences between social and non-social content, suggesting that social attention is inherently different from general attention mechanisms. This, in turn, raises the question whether a special neuro-cognitive system, distinct from the ventral or dorsal network suggested for bottom-up and top-down attention, mediates social attention and its rapid allocation. The study of Kennedy & Adolphs^14^, who showed that irregular bottom-up processing caused by amygdala lesions can be overcome by using a gaze-contingent paradigm, indicates how important the disentanglement of these processes are. Furthermore, such patient studies can offer insight to underlying mechanisms and further our understanding of brain areas involved in social processing. Future neuroimaging studies investigating potential candidates for a social attention network are necessary to further elucidate this assumption.

Even though our findings depict robust and successfully replicated results, our study has a few limitations. First, we cannot control for certain influences arising from the use of naturalistic stimuli. For instance, although the distribution of social features within the images was considered, such that they were not always presented centrally, in the foreground or depicted only single individuals, the currently used stimulus set has some variability in the specific scene composition which might reduce the internal validity of the current setup. Furthermore, even though we carefully controlled physical image properties such as feature congestion, subband entropy, edge density, and overall saliency and ensured that these measures did not differ between social and non-social images^16^, we could not control for every aspect of scene composition and structure. For example, even though spatial frequencies have repeatedly been reported to affect attentional capture^25,26^, we did not control for a similar distribution of features within specific frequency bands of the current stimulus set. Nevertheless, we chose these complex scenes as they have comparatively high ecological validity and contain contextual information which plays an important role for the orientation in our environment^6^. Moreover, we deliberately wanted to defer from isolated or artificial setups, as they include viewing conditions that do not resemble important properties of the input our visual system has to deal with every day (see^27,28^). However, it is important to note that the use of photographs of naturalistic scenes, has also been put into question^27^, as these are not equivalent to experiencing the real world and some recent studies have indeed shown conflicting results comparing eye tracking in the laboratory with to mobile eye tracking^29^. Second, our study included animal pictures in the set of non-social scenes and studies have shown that eye movements may be influenced by animacy of depicted features within a complex scene (e.g.,^30,31^). However, this theory implies that gaze behavior for our non-social stimuli should be similarly biased (e.g., by enhancing gaze towards animals) as for social stimuli. Even so, our results still show better predictions through saliency measures for non-social stimuli and higher recurrent fixations for social as compared to non-social stimuli within our recurrence quantification analysis. Therefore, social features may still be preferred even over other animate aspects. Future studies should examine this hypothesis using a balanced set of pictures either including humans and animals in the same scene or in different sets of photos. Third, the currently used stimuli might be perceived differentially regarding emotional aspects or personal relevance and these dimensions might in turn also affect exploration patterns. While we refrained from requiring stimulus ratings in the current study due to time constraints, we collected emotional valence, arousal and personal relevance ratings for the currently used stimuli in a previous study and showed that social and non-social scenes were comparable on these dimensions^16^. Since the sample of the current study was largely similar to the sample examined before (e.g., regarding age, education and health), we did not expect to find differences in subjective ratings in the current study. However, it might be interesting for future research to directly examine the influence of affective dimensions or perceived personal relevance on viewing patterns. One recent study already demonstrated an influence of emotional valence on the visual exploration of video clips^32^ and it is currently unclear whether similar effects can also be obtained for static stimuli.

In summary, this study successfully replicated and extended previous research using recurrent quantification analysis, showing that gaze patterns were not only very different for free-viewing as opposed to gaze-contingent viewing, but also for social compared to non-social content. This attention bias was also evident for fixation densities and cannot be accounted for by physical saliency. Concluding, our results imply a social prioritization that appears to involve voluntary attentional selection and thereby substantiates the notion that social stimuli are exceptional concerning visual attention.

## Methods

### Participants

We used power analyses^33^ to calculate the number of participants necessary for revealing medium-sized effects in paired t-tests (Cohen’s *d* = 0.50) or repeated measures analyses of variance (ANOVAs, *f* = 0.25), respectively, at a significance level of α = 0.05 and a power of 0.95. When assuming a correlation of *r* = 0.50 between factor levels in the ANOVA, these analyses revealed a required sample size of 54 participants. We thus aimed at recruiting a minimum of 60 participants in order to account for potential dropouts.

Since participant recruitment was more successful than anticipated, a total of 82 subjects (37 males) participated in this study. Of these 82 participants, 30 participants were recruited primarily from the University of Würzburg’s Human Participant Pool and 52 from a database allowing pre-screening of social anxiety and the subsequent selection of a normal distribution of social anxiety (which is of no further relevance to the current study). Three participants were excluded because of current medication usage or a neurological illness. Participants with more than 30% missing baseline values or outliers (see below) were also not considered in the analysis resulting in the exclusion of four additional participants. The final sample thus consisted of 75 participants (30 males) with a mean age of 24.08 years (*SD* = 5.29 years). All participants had normal or corrected-to-normal vision.

The study was approved by the by the ethics committee of the German Psychological Society (DGPs) and conducted according to the principles expressed in the Declaration of Helsinki. Each participant provided written informed consent prior to the experiment and was awarded extra course credit or monetary compensation.

### Apparatus

The experiment was programmed with MATLAB© 2011b (Mathworks, Inc., Natick, MA, USA) using the Psychophysics Toolbox (Version 3.0.12)^34–36^ and presented on an LG 24MB 65PY-B 24″ monitor with a physical display size of 516.9 × 323.1 mm. The monitor had a resolution of 1920 × 1200 pixels and a refresh rate of 60 Hz. Eye movements were tracked using a mounted EyeLink 1000 Plus system (SR Research Ltd., Ottawa, Canada). The sampling rate was set to 1000 Hz and we tracked the right eye at a viewing distance of 50 cm.

### Stimuli

The stimuli used consisted of 160 naturalistic images. Half of these images displayed scenes containing one or more human beings displayed anywhere within the image, which will be referred to as social images in the following. The other 80 images showed scenes containing non-social features, predominantly complex landscapes, including objects and on rare occasions animals. The stimulus set was taken from End and Gamer^16^ and created from various image databases including the Nencki Affective Picture System^37^, EmoPics^38^, the International Affective Picture System (IAPS^39^), McGill Cailbrated Colour Image Database^40^, Object and Semantic Images and Eye tracking dataset (OISE^41^) and websites such as Flickr and Google (selected images from databases are specified in section S3 in the Online Supplement). Contrast and luminance were adjusted manually by visual judgement. Stimuli were presented in a resolution of 1200 × 900 pixels resulting in a visual angle of 35.81° × 27.24° within the current setup. The currently used social and non-social images were already employed in a previous study and were shown to be comparable regarding basic visual properties such as image complexity or clutter as well as affective quality and personal relevance^16^.

### Design

**Figure 5:**
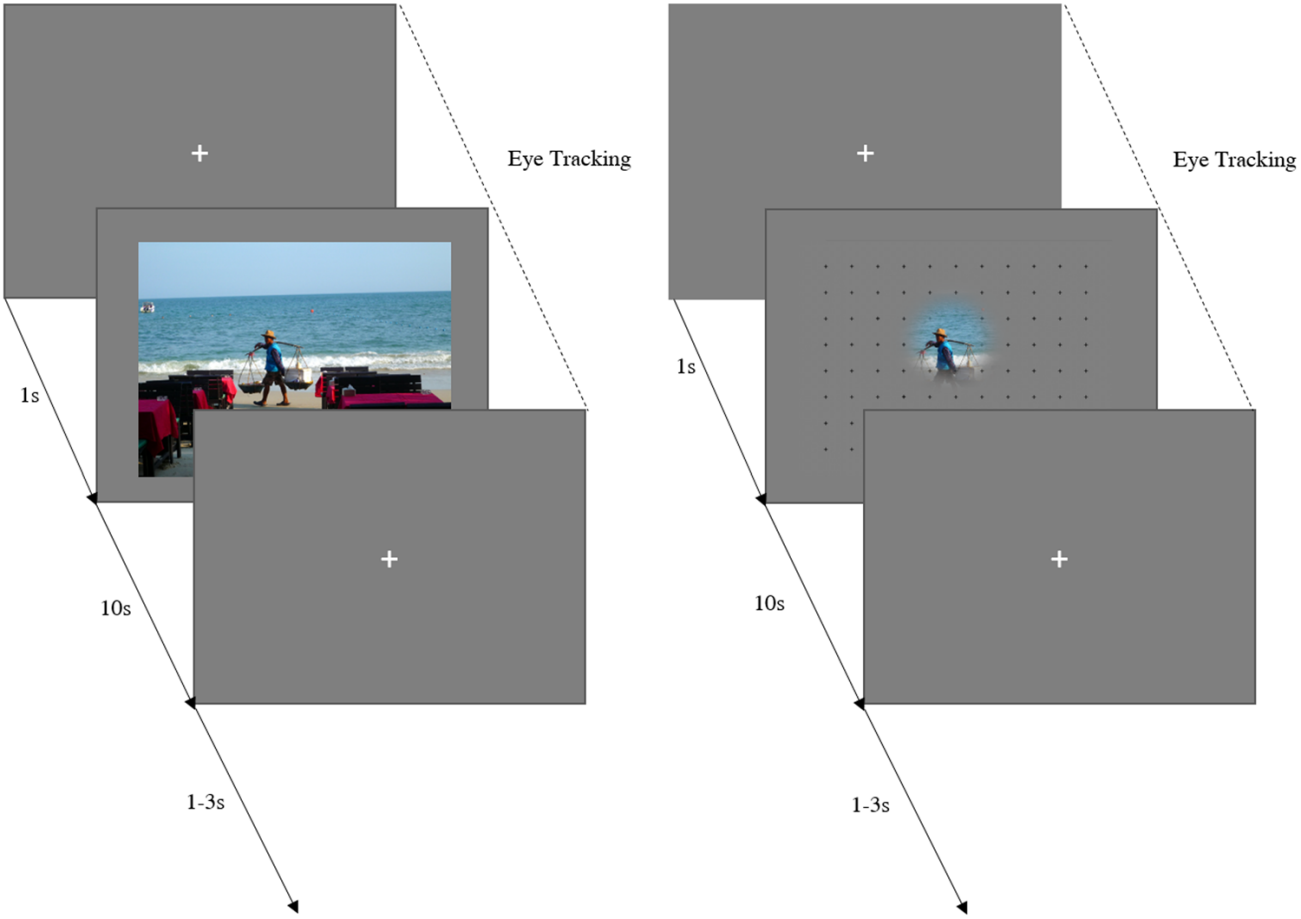
Example of an experimental trial for a free-viewing condition (left) and a gaze-contingent condition (right). The presentation time for both conditions was set to 10 s. Image taken with permission from the Nencki Affective Picture System^37^.

The experiment consisted of two different types of viewing modalities for the stimuli: (1) free-viewing and (2) gaze-contingent viewing. For each participant, images were randomly associated to these viewing conditions while ensuring for an equal number of social and non-social images in each condition. In the free-viewing condition, the whole image was visible at a time and could be explored freely. The gaze-contingent display enabled the participant to only see the part of the stimulus that was centered at the current fixation location. The online tracking enabled real-time contingency on the display with the movement of the participant’s eye. The visible area was defined by a Gaussian transparency mask with full-width half-maximum of 3° of visual angle around the center of the current fixation location (adapted from Kennedy & Adolphs^14^). The stimuli in the gaze-contingent condition were masked with a fixed grid of small dots located 2.2° from one another with a 3-pixel diameter to offer a sense of coordination during stimulus exploration (Fig. 5). There was no postulated task for either the free-viewing nor the gaze-contingent condition, but participants were instructed that they could explore the stimuli freely if desired. Further, they were informed that the image would be masked in the gaze-contingent condition and that they would be able to uncover image areas by moving their eyes.

### Procedure

Each trial began with a fixation cross presented on a grey background for 1 s. Stimuli were presented for 10 s in both viewing conditions. Afterwards, a fixation cross appeared again comprising an inter-trial-interval of 1–3 s. The experiment was divided into four different blocks, two of which were free-viewing, the other two were gaze-contingent. The blocks were alternated as such that a block of one condition would always follow a block of the other. Every second participant started with a gaze-contingent block to avoid sequence effects. A 9-point calibration was conducted at the beginning of each block and a drift correction after every 8 trials to ensure precise measurement and correct exposure of stimulus details in the gaze-contingent condition. Six training trials using a different set of pictures were included to enable participants to become acquainted with the paradigm.

### Data analysis

Data were analyzed using the open-source statistical programming language R (www.r-project.org, version 3.3.3) and MATLAB® R2011b. The R-package *ez* (version 4.3)^42^ was used for all repeated-measures analyses of variance (ANOVAs). An a priori significance level of α = 0.05 was specified for all statistical tests. Generalized η^2^ ^43^ and Cohen’s *d* are reported as estimates of the effect size for ANOVAs and *t*-tests, respectively. The Huynd-Feldt procedure was used for all repeated-measures ANOVAs containing more than one degree of freedom in the enumerator to account for potential violations of the sphericity assumption.

### Eye Tracking Preprocessing

Eye tracking data preprocessing was essentially identical to an earlier study^17^ including all commonly applied steps – drift correction, iterative baseline outlier removal and creation of fixation maps with Gaussian kernel smoothing of 2° of visual angle (see Supplementary Material S1 for full details).

### General Influence of Saliency

In order to determine to what degree low-level visual saliency predicts fixations for social and non-social scenes, we compared similarities between fixation density and saliency maps. The latter were calculated for each image using the Graph-Based Visual Saliency (GBVS) algorithm^44^ that was shown to be capable of predicting visual exploration with considerable accuracy^1,45^. Similar to fixation densities, saliency maps were normalized to range from 0 to 1. Both maps were compared using standard metrics^46^. These comprised the divergence of the distributions of physical saliency and fixation density (Kullback-Leibler divergence, *D*_*KL*_)^47,48^, the classification of saliency at fixated and non-fixated image locations (area under the receiver-operating characteristic curve; *AUC*)^49,50^ and the linear dependence between the two variables (Pearson product-moment correlation coefficient *r*)^51,52^. For AUC, fixation density maps were binarized using the mean fixation density as threshold. All metrics were calculated separately for social and non-social scenes and the two viewing conditions and compared using 2 × 2 repeated-measures ANOVAs with factors viewing condition (free-viewing, gaze-contingent) and stimulus category (social, non-social) on each measure.

### Regions of Interest

To quantify the fixation density onto physically salient aspects and social features, we introduced regions of interest (ROIs). Similar to our previous studies^16,17^, we differentiated between regions of high saliency, low saliency, head and body (see Supplementary Material S2 for full details). Area-normed fixation density scores for these ROIs were analyzed using a 2 × 4 repeated-measures ANOVA with factors viewing condition (free-viewing, gaze-contingent) and ROI (head, body, low saliency, high saliency).

To investigate potential influences on attention towards social features in the gaze-contingent viewing condition as compared to free-viewing, we conducted post hoc analyses to determine if the observed difference was due to a significant time difference in initial detection of the social feature. Hence, we compared viewing conditions anew, selecting fixations from the time point in which a social ROI (head or body) was first fixated. The time points of initial social fixations were compared for both viewing conditions in a paired *t*-test for the social stimulus set. Furthermore, we generated new fixation density maps for the time window after the social ROI was detected and analyzed area-normed fixation densities on ROIs using a 2 × 4 repeated-measures ANOVA with factors condition (free-viewing, gaze-contingent) and ROI (head, body, areas of low saliency and high saliency).

### Recurrence Quantification Analysis

Another tool for describing complex dynamic systems and characterizing gaze patterns is recurrence quantification analysis^19,53,54^. Herein, fixations which repeatedly occur at the same location can be identified, which offers additional information about gaze patterns in the presence of social features for different viewing conditions. The determination of whether a fixation was recurrent or not was accomplished by a fixed radius revolving around the previous fixations. The radius was chosen according to the size of the gaze-contingent window used in the experiment (adopted from Anderson *et al*.^19^) and thus amounted to 97 pixels, which is equivalent to 3° visual angle^53^. To compare fixation sequences across experimental conditions, quantitative measures were extracted, namely, a recurrence measure (how often observers fixate previously viewed image locations), a determinism measure (describing fixation locations that likely follow one another), a laminarity measure (indicating that regions were fixated multiple times) and a center of recurrence mass (CORM; indicates where in time most of the recurrent fixations were located with small CORM values implying re-fixations that are closer in time than those with large CORM values) (for details see Anderson *et al*.^19^; the code was kindly made available by Nicola Anderson and implemented in MATLAB). The measures were computed separately for both viewing conditions and social and non-social images and subsequently analyzed in four 2 × 2 repeated-measures ANOVAs with factors viewing condition (free-viewing, gaze-contingent) and stimulus category (social, non-social).

The datasets generated during and/or analyzed during the current study are available from the corresponding author on reasonable request.

## Electronic supplementary material

Supplementary Material
