# Supplementary material for: Attentional Selection of Social Features Persists Despite Restricted Bottom-Up Information and Affects Temporal Viewing Dynamics
Source: Sci Rep. 2018 Aug 22;8:12555. doi: 10.1038/s41598-018-30736-8 (PMC6105690; doi:10.1038/s41598-018-30736-8)
Supplement: Supplementary file 1 — Supplementary Material [file 41598_2018_30736_MOESM1_ESM.pdf]

Supplementary Material

Attentional Selection of Social Features Persists Despite Restricted Bottom-Up Information and Affects Temporal Viewing Dynamics

[1]Aleya Flechsenhar[a], [1]Lara Rösler[a]*, [1]Matthias Gamer

[1] Department of Psychology, Julius Maximilian University of Würzburg, Würzburg, Germany

[a]These authors contributed equally

*Corresponding author: lara.roesler@uni-wuerzburg.de

S1 Eye-Tracking Preprocessing Details

Saccades and were detected from the recorded eye-tracking data by using a velocity and an acceleration threshold of 30°/s or 8000°/s^2, respectively. Time periods between saccades were defined as fixations and their coordinates (x, y) and durations saved for subsequent analyses. Fixations were drift-corrected with reference to a baseline period of 300 ms during the presentation of the fixation cross directly preceding stimulus presentation. Similar to previous studies, fixations that deviated from this baseline were identified by a recursive outlier removal procedure that was applied separately to x- and y-baseline-coordinates (see [1–3]). In detail, this procedure temporarily removed the highest and lowest coordinates for each participant from the baseline distribution and compared it to the mean and standard deviation of the remaining data. If these values were more than three standard deviations below or above this mean, they were marked as outliers, otherwise, they were returned to the distribution. This procedure was repeated until no more values were defined as outliers. Baseline outliers or missing baseline coordinates (social scene trials: M = 5.89%, SD = 6.15%; non-social scene trials: M = 4.78%, SD = 4.73%) were replaced with the mean baseline position of all scenes with valid baseline position data of the respective participant.

Following baseline correction of all fixations within each trial, a fixation density map was created by storing fixation coordinates in an empty matrix with the same dimensions as the currently used stimuli (1200 x 900 pixels). Fixations were weighted by their duration in ms. The resulting map was smoothed with an isotropic Gaussian kernel with a standard deviation of 32 pixels corresponding to 1° visual angle in positive and negative direction using the R package *spatstat* [4] (version 1.47.0). The resulting 2° of visual angle correspond to the functional field of the human fovea centralis. In a final step, the fixation density maps were normalized to values between 0 and 1.

S2 Region of Interest Details

Saliency maps were used to identify regions of high saliency (above the eighth percentile of the saliency map) and areas of low saliency (below the eighth percentile) for all stimuli. Additionally, we manually defined regions for head and body of depicted human beings for social scenes using the software GNU Image Manipulation Program (GIMP; Version 2.8.10). A ROI could only be defined once, so that areas

of high and low saliency for social scenes were restricted to those that had not yet been defined by head and body ROIs. In a previous study, we already demonstrated that social ROIs (head and body) had a lower mean saliency than highly salient non-social image regions for this stimulus set[1]. To determine the extent to which each ROI was fixated by the participant, we calculated the sum of fixation density values for each ROI and divided it by the sum of fixation density values for the whole stimulus. To take into account the different sizes of ROIs, this proportion was then normalized by dividing it by the area of the ROI. These area-normed fixation density scores were analyzed using a 2 x 4 repeated-measures ANOVA with factors viewing condition (free-viewing, gaze-contingent) and ROI (head, body, low saliency, high saliency).

S3 References of Stimuli from Databases

Stimuli taken from different databases (*n* = 67) with according reference and content differentiation for this study. The remaining stimuli (*n* = 93) were taken from internet sources (e.g., Google, Flickr etc.)

**Database**	**Reference**	**Content**
Emotional Picture Set	9.jpg	social
Emotional Picture Set	119.jpg	social
Emotional Picture Set	131.jpg	social
Emotional Picture Set	133.jpg	social
Emotional Picture Set	138.jpg	social
Emotional Picture Set	191.jpg	social
Emotional Picture Set	196.jpg	social
Emotional Picture Set	197.jpg	social
Emotional Picture Set	205.jpg	social
Emotional Picture Set	267.jpg	non-social
Emotional Picture Set	280.jpg	non-social
International Affective Picture System	5199.jpg	social
International Affective Picture System	9150.jpg	social
International Affective Picture System	9186.jpg	non-social
International Affective Picture System	9422.jpg	non-social
McGill Calibrated Colour Image Database	Merry_0005_Lasalle.jpg	non-social
McGill Calibrated Colour Image Database	Merry_0014_Lasalle.jpg	non-social
McGill Calibrated Colour Image Database	Merry_0060_Lasalle.jpg	non-social
McGill Calibrated Colour Image Database	Merry_0064_Lasalle.jpg	non-social
McGill Calibrated Colour Image Database	Merry_florida0011.jpg	social
McGill Calibrated Colour Image Database	Merry_florida0017.jpg	non-social
McGill Calibrated Colour Image Database	Merry_mexico0072.jpg	social
McGill Calibrated Colour Image Database	Merry_mexico0143.jpg	social
McGill Calibrated Colour Image Database	Merry_0081.jpg	non-social

McGill Calibrated Colour Image Database	Pippin_city6.jpg	social
McGill Calibrated Colour Image Database	Pippin_city66.jpg	social
Nencki Affective Picture System	Animals_025.jpg	non-social
Nencki Affective Picture System	Animals_048_h.jpg	non-social
Nencki Affective Picture System	Animals_074_h.jpg	non-social
Nencki Affective Picture System	Animals_102_h.jpg	non-social
Nencki Affective Picture System	Animals_128_h.jpg	non-social
Nencki Affective Picture System	Animals_194_h.jpg	non-social
Nencki Affective Picture System	Animals_195_h.jpg	non-social
Nencki Affective Picture System	Animals_201_h.jpg	non-social
Nencki Affective Picture System	Animals_218_h.jpg	non-social
Nencki Affective Picture System	Faces_023_h.jpg	social
Nencki Affective Picture System	Faces_265_h.jpg	social
Nencki Affective Picture System	Faces_290_h.jpg	social
Nencki Affective Picture System	Faces_302_h.jpg	social
Nencki Affective Picture System	Landscapes_016_h.jpg	non-social
Nencki Affective Picture System	Landscapes_025_h.jpg	non-social
Nencki Affective Picture System	Landscapes_040_h.jpg	non-social
Nencki Affective Picture System	Landscapes_043_h.jpg	non-social
Nencki Affective Picture System	Landscapes_064_h.jpg	non-social
Nencki Affective Picture System	Landscapes_071_h.jpg	non-social
Nencki Affective Picture System	Landscapes_085_h.jpg	non-social
Nencki Affective Picture System	Landscapes_178_h.jpg	non-social
Nencki Affective Picture System	Objects_002_h.jpg	non-social
Nencki Affective Picture System	Objects_013_h.jpg	non-social
Nencki Affective Picture System	Objects_058_h.jpg	non-social
Nencki Affective Picture System	Objects_183_h.jpg	non-social
Nencki Affective Picture System	Objects_202_h.jpg	non-social
Nencki Affective Picture System	Objects_214_h.jpg	non-social
Nencki Affective Picture System	People_009_h.jpg	social
Nencki Affective Picture System	People_015_h.jpg	social
Nencki Affective Picture System	People_022_h.jpg	social
Nencki Affective Picture System	People_054_h.jpg	social
Nencki Affective Picture System	People_058_h.jpg	social
Nencki Affective Picture System	People_109_h.jpg	social
Nencki Affective Picture System	People_116_h.jpg	social
Nencki Affective Picture System	People_131_h.jpg	social
Nencki Affective Picture System	People_157_h.jpg	social
Nencki Affective Picture System	People_158_h.jpg	social
Nencki Affective Picture System	People_167_h.jpg	social
Nencki Affective Picture System	People_182_h.jpg	social
Nencki Affective Picture System	People_195_h.jpg	social
Object and Semantic Images and Eyetracking dataset	118.jpg	non-social

Supplemental References

1. End, A. & Gamer, M. Preferential processing of social features and their interplay with physical saliency in complex naturalistic scenes. *Front. Psychol.* **8,** 418 (2017).

2. Flechsenhar, A. F. & Gamer, M. Top-down influence on gaze patterns in the presence of social features. *PLoS One* **12,** 1–20 (2017).

3. Rösler, L., End, A. & Gamer, M. Orienting towards social features in naturalistic scenes is reflexive. *PLoS One* **12,** e0182037 (2017).

4. Baddeley, A., Rubak, E. & Turner, R. Spatial Point Patterns: Methodology and Applications with R. (2015).
